# Use of Muscle Interpolation Flap as a Reconstructive Option in Soft Tissue Coverage of Limbs: A Novel Technique

**DOI:** 10.1055/s-0045-1802629

**Published:** 2025-02-12

**Authors:** Bharatendu Swain, Shalini Sampreethi

**Affiliations:** 1 Department of Plastic and Reconstructive Surgery, Aakar Asha Hospital, Kukatpally, Hyderabad, Telangana, India

**Keywords:** interpolation muscle flap, vascular compromise, hand reconstruction, heel reconstruction

## Abstract

**Background:**

In injuries of the hand or forearm with vascular compromise due to a single vessel supplying blood distally or difficulty in donor vessel access, a pedicled flap is preferred. Skin flaps like the groin flap are commonly used as an interpolation flap. Muscle flaps used as interpolation flaps have scarcely been reported in the literature. However, muscle has been a component of composite flaps used as interpolation flaps like the tongue flap. The authors have used pedicled, interpolated muscle flaps successfully in hand and heel reconstructions.

**Materials and Methods:**

Five patients with soft tissue loss due to trauma, in single vessel limbs or difficult donor vessel access, were taken up for interpolation muscle flap. Four of these patients underwent inferiorly based, upper rectus abdominis muscle flap delivered at the umbilicus, by endoscopy or the open method. One patient underwent a proximally based, medial head of the gastrocnemius, cross-leg flap. The muscle flaps were skin grafted. One month later, the flaps were delayed and divided shortly thereafter. The divided end of the muscle was dressed till it healed. All the patients were followed up for healing time, additional procedures, and long-term results.

**Results:**

Five documented cases of reconstruction in single vessel limbs or difficult donor vessel access were reviewed. All five cases achieved good healing and intended reconstructive outcomes without any complications.

**Conclusion:**

An interpolation muscle flap is a safe and effective method for reconstructing limb defects. Both types of interpolation muscle flaps described are novel applications in reconstructive surgery.

## Introduction

Knowledge of microsurgery in the late 1980s and perforator vessel anatomy in the first decade of this century turned the tide in favor of free tissue transfers and perforator flaps, respectively, for reconstruction of limb defects. There are situations, however, where a single artery blood supply or unfavorable soft tissue conditions make it difficult to execute safe and effective free tissue transfer or locoregional flaps. In such situations, an interpolation flap is considered, such as the groin or cross-leg flap, which transfers healthy tissue to the defect. Muscle flaps have rarely been used in isolation as interpolation flaps. This innovative case series demonstrates that interpolation muscle flaps can be used safely and effectively for soft tissue cover of a limb defect, even facilitating bone graft healing. The upper half of the inferiorly based rectus muscle and the medial head of the gastrocnemius muscle were successfully used as interpolation flaps.

## Materials and Methods

Medical records of five patients who had undergone muscle interpolation flap for reconstructing limb defects following trauma were obtained.

All the patients had a single artery perfused limb or difficult donor vessel access, without adequate healthy locoregional tissue. Four of these patients had upper limb soft tissue deficits and one had an adherent skin grafted heel that precluded heel walking.

The patients or parents were counseled regarding the options for surgery, and the decision to do the interpolation muscle flap was made as per their preference.

All the patients were worked up preoperatively with laboratory tests and X-rays. Surgery was done under general anesthesia.

Patient demographic and clinical details, operating time, cumulative hospital stay, healing time, secondary procedures, and outcomes were noted ( Tables 1 and 2 ).

**Table 1:** Patient details

Sl. no.	Age (y)	Sex	Cause of injury	Previous surgery	Injury–muscle flap interval	Clinical details
1	4	Male	Circular saw used for cutting ice bricks	None (acute injury)	11 d	Right mid-metacarpal, 4-digit amputation replanted; loss of 2 digits and exposure of all metacarpal heads
2	23	Male	Motor bike accident	None (acute injury)	10 d	Crush injury with segmental loss of ulnar vessels; loss of right first metacarpal with dorsal skin loss of the thumb and first web
3	26	Male	Sugarcane crush injury	Debridement and groin flap cover were done	8 mo	Status post groin flap coverage after crush amputation of all digits at the base proximal phalanx level
4	24	Male	Electrical injury	Debridement	5 mo	Electrical injury left forearm with large segment of the exposed devitalized radius bone
5	14	Female	Vehicle run over injury in a road accident	Bony fixation and skin grafting of the right foot including heel.	1 y	Adherent skin covered heel with circumferential foot graft

**Table 2:** Surgical and post-surgical details

Sl. no.	Flap details	Adjunct procedures	Later procedure(s)	Duration of surgery	Cumulative hospital stay	Healing time	Follow-up period	Outcome
1	Endoscopic dissection of the upper half of the right rectus abdominis	Skin grafting	None	2 h and 45 min	3 wk	6 wk	12 y	Uses the hand for support
2	Endoscopic dissection of the upper half of the right rectus abdominis	External fixator spacing; skin grafting	Bone graft for the 1st metacarpal; silicon interposition arthroplasty at the base of the 1st metacarpal	3 h and 15 min	15 d	5 wk	8 y	Writes with the right hand and uses the thumb for all grip functions
3	Combined old scar and short incision access for harvest of the upper rectus abdominis flap	Bone grafting of the proximal phalanx bases; skin grafting	None	4 h and 20 min	18 d	10 wk	2 y	Using the right hand for gripping objects
4	Open harvest of the left rectus abdominis flap	Bony debridement; skin grafting	Vascularized bone graft (done elsewhere)	2 h and 10 min	21 d	7 wk	1 y	Using the left hand for all activities
5	Cross-leg medial gastrocnemius muscle flap	Removal of adherent skin graft from heel; skin grafting	None	3 h 10 and min	50 d	10 wk	1.5 y	Able to walk 1 km to school

For upper limb reconstruction, the ipsilateral rectus abdominis muscle was detached from its insertion to the ribs and dissected down to the umbilicus, by endoscopy or open method, and delivered at the umbilicus. The distal part of the muscle was inset to the margins of the defect and covered by a skin graft. The upper limb was immobilized by adhesive strapping to the chest as in a groin flap.

For heel reconstruction, the contralateral medial head of the gastrocnemius muscle was raised, proximally based, by a vertical posteromedial skin incision, inset transversely to the margins of the heel defect, after excision of adherent heel skin graft and covered by a skin graft. External fixators were applied to prevent disruptive movement of the cross-leg muscle flap.

After a 1-month delay, the muscle flaps were done and divided 2 days later. The donor site was closed directly. The upper limb was immobilized by adhesive strapping to the chest as in a groin flap.

Ancillary bony procedures were performed simultaneously in one case and in a second stage in two cases.

## Results

Five patients underwent the muscle interpolation flap. Four patients had upper limb soft tissue defects. One case was a 4-year-old with a 12-hour-old traumatic crush amputation of the right hand at the mid-metacarpal level. He underwent replantation of four digits and suffered postreplantation loss of two digits at the metacarpophalangeal joint level and exposure of all four metacarpal heads. The second case was of a traumatic first metacarpal loss and large raw area spanning the first web space dorsum. 1 The third case was a postoperative case of crush amputation of the right hand at the level of the proximal phalangeal bases, covered with a groin flap, done elsewhere. The fourth case was an electrical injury of a single remaining upper limb with exposed devitalized radius bone. The fifth case was one of heel loss in a circumferential avulsion of the foot and ankle, skin grafted earlier.

Patient ages ranged between 4 and 26 years, with an average age of 18.2 years. All patients were followed up for periods ranging from 1 to 12 years, with an average follow-up of 4 years and 9 months. The average duration of surgery was 3 hours and 8 minutes and ranged from 2 hours and 10 minutes to 4 hours 20 minutes for the entire procedure. Hospital stays ranged from 15 to 50 days, averaging 25 days for the given episode. Healing time was taken from the date of surgery till the divided muscle stump healed at the site of reconstruction and ranged from 6 to 10 weeks, averaging 7.5 weeks. The patients were followed up at regular intervals. The reconstructive goals were achieved in all cases without any major complications. The divided muscle end suffered a short segmental necrosis, which was treated conservatively with dressings till healing in four cases and was skin grafted in one case.

**Fig. 3:**
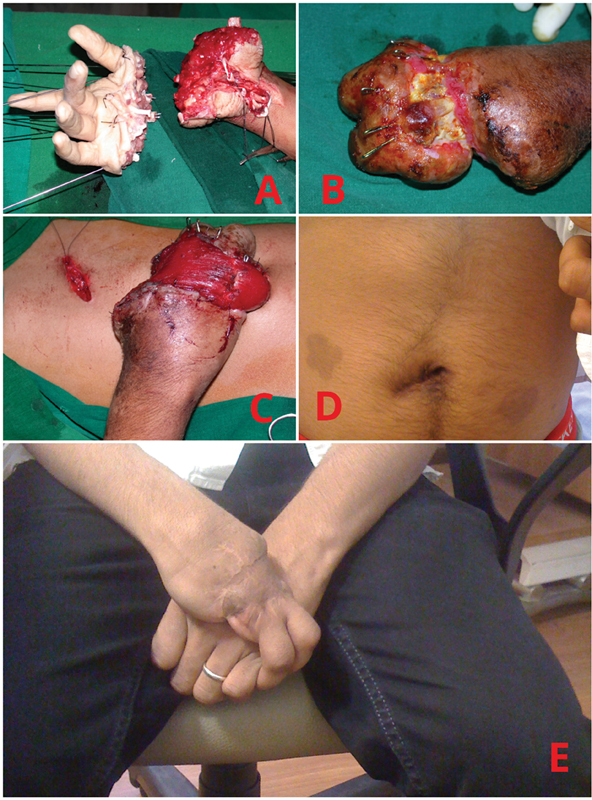
Case 3. ( **A** ) Preoperative picture of skin grafted right foot with missing heel. ( **B** ) Cross-leg muscle flap with skin graft. ( **C** ) Division of the pedicle. ( **D, E** ) Postoperative images of the reconstructed heel.

There was no abdominal wall hernia in any of the four cases of rectus abdominis muscle transfer or leg weakness in the case of the transfer of the medial head of the gastrocnemius muscle. As both anterior and posterior rectus sheaths are retained and the residual inferior half of the rectus innervated, postoperative herniation is unlikely. Three of the four upper limb cases had an additional bony procedure. The periumbilical and upper abdominal short scars especially with endoscopic harvest are not very prominent ( Fig. 3D ). Groin flap scars can sometimes stretch and become unsightly.

Three case reports are detailed below.

### Case 1

**Fig. 1:**
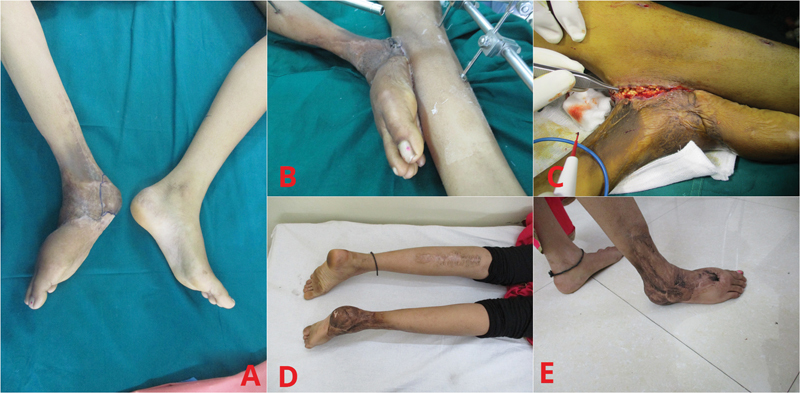
Case 1. ( **A** ) Amputated fingers of the right hand. ( **B** ) Exposedmetacarpal heads. ( **C** ) Pedicled interpolation upper rectusmuscle flap. ( **D** ) Donor site after 10 years. ( **E** ) Healed hand with intact two fingers 10 years later.

A 14-year-old adolescent girl suffered circumferential degloving injury of her right foot and fracture of her right tibia in a vehicle runover injury a year earlier. The foot wounds had been skin grafted and bony fixation done ( Fig. 1A ). She presented to us a year later with the inability to walk on her right heel. An earlier angiogram had reported flow through the right peroneal artery and a biphasic flow through the posterior tibial artery. Regional flaps like Reverse Sural Artery (RSA) and cross-leg fasciocutaneous flaps were considered but not used as they can be wobbly and unstable while walking. Free muscle transfer with skin graft is preferred. In view of the dense scarring of the medial and anterior lower leg making vessel access difficult, she underwent a cross-leg flap of the medial head of the left gastrocnemius muscle after removing the adhered skin graft of her right heel. The muscle flap was inset to the heel defect. The position of the right heel was maintained with external fixation ( Fig. 1B ). A month later, the flap was divided after delay ( Fig. 1C ) and external fixation dismantled. The stump healed in 3 weeks. Gradual ambulation was commenced after a month and complete weight bearing was achieved after 5 months ( Fig. 1D and E ). At 1.5 years, she increased her walking distance, walking rapidly with good heel contact and no discomfort, for a kilometer or more to school.

### Case 2

A 30-year-old man presented with a previously done groin flap covering thin bases of the proximal phalanges of his right hand, preserving the metacarpophalangeal joints and an intact thumb with a shortened tip.

**Fig. 2:**
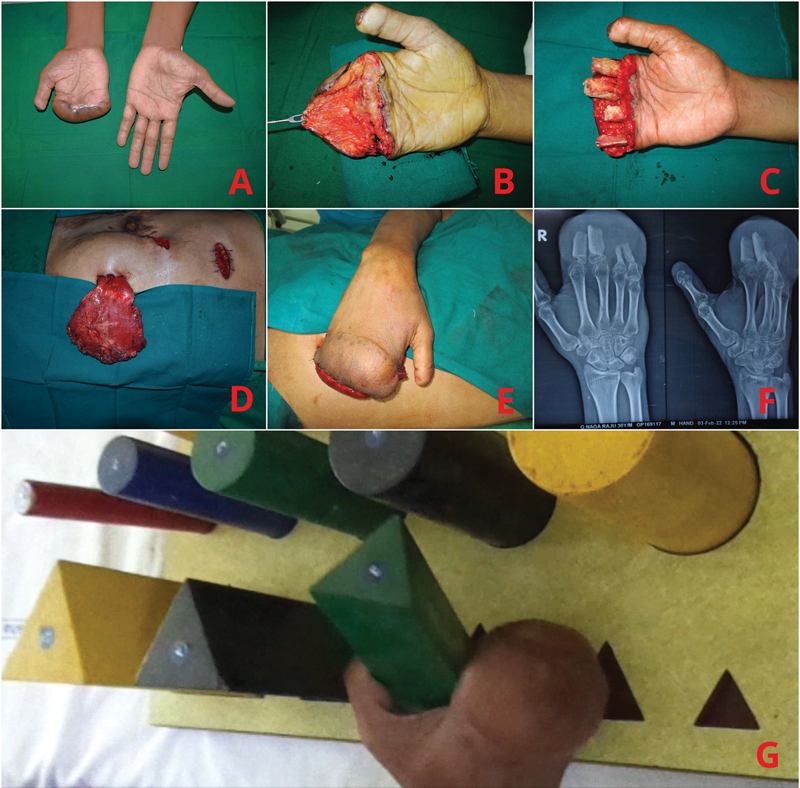
Case 2. ( **A** ) Amputated fingers of the right hand with previous groin flap. ( **B** ) Unfurled groin flap. ( **C** ) Bone grafts attached to the proximal phalanx stumps. ( **D** ) Pedicled upper rectus muscle flap. ( **E** ) Flap attached. ( **F** ) Six-month postoperative X-rays. ( **G** ) Longer stump enabling him to grasp large objects.

He had sustained amputation of his dominant right hand in a sugarcane crushing machine; debridement and groin flap cover had been done 6 months earlier ( Fig. 2A ).

As he was unwilling to undergo a toe transfer, elongation of the bony stumps and extension of the soft tissue cover were planned in one procedure to give him a better functioning hand. The bony stumps were planned to be individually elongated with bone grafts to keep the possibility of separating them at a later date.

The groin flap over the right hand stump was opened in a dorsally based manner, leaving a large volar defect ( Fig. 2B ). In view of his existing abdominal scars, additional umbilical and small vertical upper abdominal incisions for access were made. The upper half of the right rectus abdominis muscle was dissected and delivered through the umbilical incision ( Fig. 2D ). Through a right hip incision, the iliac crest was exposed, and a large inner table bone graft was harvested. Bone graft segments of 5 to 6 cm were taken from the main piece. The bone segments were fixed to the open ends of the existing proximal phalangeal bases with 1-mm Kirschner's wires ( Fig. 2C ). The distal part of the rectus muscle was draped over the bony ends and secured with 3–0 Vicryl (Ethicon, India). The muscle was covered with a split-thickness skin graft (SSG; Fig. 2E ).

One month later, the flap base was delayed and then divided after 48 hours. The large open end of the inset flap was left to heal for 3 weeks, after which the granulating surface was covered with an SSG.

Six months later, he had healed bone grafts ( Fig. 2F ) and a longer right hand stump with which he was able to get back to making sugarcane juice in a crusher for a living. He was able to use his right hand in some activities of daily living ( Fig. 2G ).

### Case 3

A 4-year-old boy sustained amputation at the mid-metacarpal level of all phalanges and thumb in a large circular saw used for cutting ice bricks ( Fig. 3A ). The amputated hand preserved in water, presented after 12 hours, was taken up for reimplantation. However, after 4 days of replantation, two of the fingers necrosed along with the entire replanted dorsal skin resulting in exposed metacarpophalangeal joints and metacarpal heads ( Fig. 3B ). In view of the distant vascular access and orientation of the defect, as well as ill-defined skin edges, the right upper rectus muscle was harvested by the endoscopic method and inset into the raw area, as a novel technique ( Fig. 3C ). The exposed bones survived and wounds healed well. Long-term follow-up showed a sleek appearance of the flap-covered areas. At 14 years of age, his family did not desire any further surgical procedure. He does not have any hernia; there is, however, a deviation of the midline ( Fig. 3D ). He uses both the remaining fingers to pick up bags and other heavy objects in a hook grip ( Fig. 3E ).

## Discussion

Limb defects are covered by a variety of locoregional flaps and free tissue transfers, including muscle flaps. Muscle flaps bring in robust vascularity, enabling bony coverage and healing, and help reduce wound infection. 2

In posttraumatic situations, with a single remaining perfusing artery and damaged or scarred surrounding soft tissue, a free tissue transfer may not succeed. In such situations, an interpolation skin or fasciocutaneous flap is usually considered.

In this series, other reconstructive options including latissimus dorsi flap had been considered but not chosen in view of the clinical situation as in the case of the patient with electrical injury of the left forearm who had extensive lower abdomen scarring following a previous groin flap.

The use of a muscle interpolation flap has not been well documented in the literature. There is just one isolated case report of a superiorly based rectus muscle flap used to cover an exposed elbow joint. 3

Flap delay in muscle has been well studied. It is well established that surgical delay is effective in augmenting the vascularity of cutaneous and myocutaneous flaps. Taylor et al 4 and Callegari et al 5 performed extensive clinical and experimental studies on the delay phenomenon and concluded that the opening or dilation of interconnecting “choke vessels” between two adjacent angiosomes following division of the dominant source artery is the main effect of surgical delay. Similar studies on muscle flap delay have been done. 6 7

In this series, the author has successfully used muscle interpolation flaps for reconstruction of defects in the upper and lower limbs, with complete healing and without any complication in all of the five documented cases. Two novel muscle flap applications have been described.

In four of the five cases involving reconstruction of upper limb defects, an inferiorly based upper half of the rectus abdominis muscle flap, delivered at the umbilicus, was used as an interpolation flap. The flap provided sleek coverage of the defect and facilitated bone grafting in two cases and bone salvage in one case. There was no donor site complication in the four cases as the lower half of the rectus abdominis had been spared ( Fig. 3D ). Hernia following entire rectus muscle harvest is mostly confined to Hesselbach's triangle.

In the fifth case, the contralateral medial head of the gastrocnemius muscle, covered by skin graft, was used for heel reconstruction enabling the patient to walk normally at long-term follow-up.

One of the apprehensions of a muscle flap after division is one of residual vascularity as reported in the literature for free muscle transfers that necrosed many years after vascular pedicle division. In a pedicled muscle flap, there is no dominant vascular supply and a substantial period of vascular delay, which account for its robust vascularity in all cases especially where bone grafting and survival were involved.

One criticism of the pedicled muscle flap has been one of inadequate pedicle length. The mobility of the upper rectus muscle and placement at the umbilicus contributed to patient comfort in four of the five cases over different age ranges.

Muscle flaps undergo denervation atrophy after division. Hence, they do not require much flap thinning.

The reconstructive goals were achieved in all cases.

## Conclusion

The interpolation muscle flap is yet another flap that can be added to the reconstructive surgeon's armamentarium of flaps. The pedicled and interpolated, inferiorly based, upper rectus abdominis muscle may be a good alternative to the groin flap especially for bony coverage and for simultaneous or later bone grafting. The access scars are less visible especially with the endoscopic approach. It can also be used for lower limb defects. With increasing experience, the indications for the interpolation muscle flap will evolve.
